# Selective laser melting of low-alloyed titanium based alloy with a large solidification range

**DOI:** 10.1016/j.heliyon.2024.e25513

**Published:** 2024-02-01

**Authors:** V.A. Bautin, V. Yu Zadorozhnyy, A.A. Korol, V.E. Bazhenov, A.S. Shinkarev, S.V. Chernyshikhin, D.O. Moskovskikh, M.E. Samoshina, A. Khort

**Affiliations:** a National University of Science and Technology MISIS, 119049, Moscow, Russian Federation; b KTH Royal Institute of Technology, SE-100 44, Stockholm, Sweden

**Keywords:** Ti-based alloy, Thermo-Calc, Powder atomization, Selective laser melting, Hot cracks, Non-equilibrium phase

## Abstract

In this work, thermodynamic calculations for α + β Type Ti–Fe–Cu–Sn alloy were carried out by the Thermo-Calc software. Powders from this alloy were obtained by plasma sputtering and used for subsequent 3D printing of experimental samples. The effect of various selective laser melting (SLM) parameters on porosity and hot cracking susceptibility as well as the electrochemical characteristics of the alloy have been studied. The optimal technological regime for the manufacture of samples by the SLM method was determined. It has been established that to obtain relatively dense samples without cracks, regimes with volumetric energy density E_v_ = 250–300 J/mm^3^ are required. It has been established that a change in the electrochemical behavior of the Ti_94_Fe_1_Cu_1_Sn_4_ alloy is related to the formation of a nonequilibrium Ti_2_Cu phase. Based on the findings we recomended directions for further research.

## 1 Introduction

Since the 1950s, joint arthroplasty has entered clinical practice and has become widespread since the early 2000s owing to the high-tech nature of this surgical intervention [[1], [2], [3]]. The materials from which modern joint endoprostheses are made have high strength and good survival rate in the human body. Therefore, their service life is on average 15–20 years, and in many cases, patients use them for up to 30 years [[4], [5], [6]]. When the endoprosthesis is worn out, it is replaced with a new one. According to patient observations, the following problems were identified in hip arthroplasty, in particular: there are massive zones of bone resorption around the acetabular component of the endoprosthesis; threads on the cavity during the operation destroy the bone tissue that goes along with osteolysis, ahead of the osseointegration of the acetabulum; and implant migration [7,8]. The main reason for such problems is the simple topology of the endoprosthesis. Since, in normal cases, a computer selection of endoprostheses takes place in a data bank with more than 40,000 prostheses, without taking into account patients’ physiological characteristics, i.e., the surgeon has to navigate the operation, which also increases the time of prosthetics and is accompanied by corresponding risks [[9], [10], [11], [12], [13], [14]].

The development of new materials with required properties for implants of the human musculoskeletal system is extremely relevant today [15,16]. An extremely large number of Ti beta-alloys have been created and studied to obtain a low modulus of elasticity [17]. Biomedical alloys have satisfactory mechanical properties and a record low modulus of elasticity. However, the existing beta alloys have a number of disadvantages [18,19]. The high content of expensive alloying elements, high density of the alloys, complex process of thermal and thermomechanical treatment, and impossibility of mechanical hardening significantly reduces the potential of these alloys [20,21]. An alpha-beta titanium alloy Ti_94_Fe_1_Cu_1_Sn_4_, reported in our previous work [22], is an alternative candidate for a new biomedical alloy. High mechanical and corrosion properties of this alloy have been also confirmed in another our previous study [23]. This paper proposes modes for obtaining spherical powder and selective laser melting (SLM) of samples from the resulting alloy powder. In addition, it presents preliminary electrochemical comparative studies and the study of the morphology of the samples’ structure. The relevance of this work is predetermined by the practical obvious possibility of using this titanium alloy as a basis for personalized implants created using additive technologies to replace old-generation orthopedic implants. Selective laser melting is a modern method of the fabrication of fully dense parts with complex shapes. This method has several advantages due to its melting peculiarities: the small size of the molten pool, short lifetime of the liquid state, and the rapid heating and cooling rates, which gives effect on superfine microstructures formation after SLM. It should also be noted that this alloy does not contain alloying components provoking allergic reactions, which will potentially have a positive effect on reducing revision operations. The investigation of the Ti-based alloys with the close composition were published in the works [[24], [25], [26]], presented including the authors of the present work.

## 2 Materials and methods

Pure metals with 99.9 % purity were used as raw materials for alloy preparation. An Arc 200 vacuum arc furnace (Arcast Inc.) with a non-consumable tungsten electrode and copper crucible at an argon pressure of 3.5·10^4^ Pa followed by purification using a Ti getter was provided for the melting operation. Five times remelting and casting into a copper mold of 120 mm in length and 14 mm in diameter was provided to obtain of the compositional homogenization for each sample. A CHMER GX-320L electric spark wire-cutting machine was used to obtain a rod samples with the following dimensions: diameter 9.5 mm; length 100 mm, for subsequent dispersion up to the powder condition.

In this work, we used the Ti_94_Fe_1_Cu_1_Sn_4_ powder with a spherical morphology and a fraction of 15–60 μm sputtered on an ATO Lab plasma ultrasonic atomizer. The rod sample was placed in a holder and then a vacuum pump was turned on to remove air before starting the spraying process, which provided high purity, i.e., less than 0.01 % O (oxygen). The argon purge mode and parameters were set: a) emitter power in a range of 240 W at an operating frequency of 40 kHz; b) plasma current from 60 A; c) argon consumption 10 l/min.

**Fig. 1:**
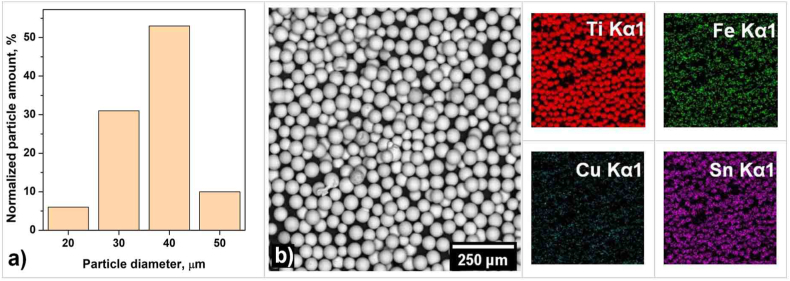
Granulometric composition (a) and the morphology of the Ti_94_Fe_1_Cu_1_Sn_4_ powder (b).

The atomized titanium alloy powder was preliminarily screened through a 0.063 mm sieve. The granulometric composition (Fig. 1a) and the morphology of the powder (Fig. 1b) have been investigated via scanning electron microscopy.

The surface morphology of the Ti_94_Fe_1_Cu_1_Sn_4_ alloy was studied using a Tescan Vega SBH3 scanning electron microscope (SEM) (Tescan, Brno, Czech Republic) equipped with an Oxford Instruments AZtecEnergy energy dispersive X-ray spectroscopy (EDS) system.

**Fig. 2:**
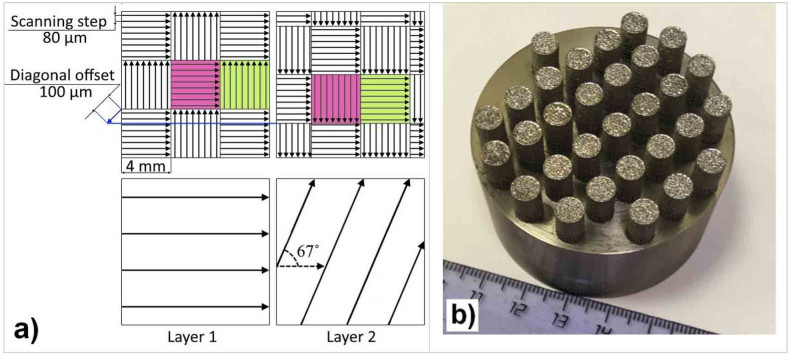
Scheme of the scanning strategy (a) and the image of the construction area with the experimental samples (b).

Samples were fabricated on an AddSol D50 setup (AddSol, Moscow, Russia) equipped with a YLR-400-WC fiber laser (IPG Photonics, Russia) at a wavelength of 1070 nm, a Gaussian distribution spot with a diameter of 80 μm, and a construction volume represented by a cylinder with a diameter of 50 mm and a height of 150 mm. The samples were printed in a protective argon atmosphere (with a residual oxygen content of less than 100 ppm) at various SLM process parameters: at laser powers of 120, 150, 250 W and scanning speed with a step of 50 mm/s in a range from 350 to 800 mm/s for each laser power values, which corresponds to the intervals of typical modes for printing titanium alloys [27,28]. The remaining parameters were constant and the following: layer thickness 30 μm, hatching step 80 μm, laser scanning strategy in a checkerboard pattern with a rotation of 67° layer by layer (Fig. 2a). The slicing procedure and executive files preparation was done with Glicer build processor (ATSS, Russia).

Each fusion mode corresponded to its own sample with a diameter of 5 mm and a height of 10 mm. The total number of samples in the construction area of 30 pieces is shown in Fig. 2b. The chemical composition of the initial alloy, powder after sputtering, as well as after SLM at volumetric energy density E_v_ = 297.62 J/mm^3^ is presented in Table 1. The chemical compositions were analyzed using energy-dispersive X-ray spectroscopy (EDS) on the three areas with a size of 1 × 1 mm^2^.

**Table 1:** Chemical composition of the Ti_94_Fe_1_Cu_1_Sn_4_ alloy.

Alloy Ti_94_Fe_1_Cu_1_Sn_4_	Content, at.%			
	Ti	Fe	Cu	Sn
As-cast	93.87	0.95	0.94	4.24
Powder	93.8	0.98	1.01	4.21
SLM E_v_ = 297.62 J/mm^3^	93.78	1.01	0.98	4.23

The thermodynamic calculations were performed using Thermo-Calc Software [29] with version 3.0 of the TTTI3 ThermoTech Ti-based alloy database [30].

To determine the porosity and conduct electrochemical studies, thin sample sections with a diameter of 5 mm each were prepared. The samples were prepared on an AutoMet 250 grinding and polishing machine (Buehler). The grinding was carried out with water cooling on magnetic disks with glued sandpaper of various grain sizes (grain distribution 120, 240, 360, 600, 1200, 2500). Polishing was carried out in two stages without the use of water cooling. At the first stage, polishing took place on a VelTex-type velvet cloth disk using diamond (MetaDi Supreme, Poly type) emulsion (abrasive particle size 3 μm) and oil (MetaDi Fluid type). In the second step, a WhiteFelt™ cloth disk was used with an Al_2_O_3_ slurry of the MicroPolish Alumina type (0.05 μm grit size) and a small amount of water.

The phase composition was studied on a DRON x-ray diffraction (XRD) instrument (Research and production enterprise "Bourevestnik", Saint Petersburg, Russian Federation) using CuKα radiation (λ = 1.5406 Å) in the range of 2θ: 30–80°. The XRD measurements in the θ–2θ stepwise scanning mode (step: 0.1°, exposition time: 5 s) were provided [31,32].

The electrochemical corrosion behavior of Ti alloys samples was performed using the IPC Pro MF (NTF Volta LLC; location: NUST MISIS) potentiostat in Hank's Balanced Salt Solution (HBSS) at 37 °C. The samples' working area was calculated using ImageJ Software. HBSS (volume of 1L) contains 8 g NaCl, 400 mg KCl, 140 mg CaCl_2_, 100 mg MgSO_4_·7H_2_O, 100 mg MgCl_2_·6H_2_O, 120 mg Na_2_HPO_4_·12H_2_O, 60 mg KH_2_PO_4_, 1 g D-Glucose, 350 mg NaHCO_3_. A three-electrode system was employed in which the samples served as a working electrode. Platinum and Ag/AgCl electrodes were used as a counter and reference electrodes, respectively. The potentiodynamic polarization curve was measured from the cathodic to the anodic region with a scan rate of 1 mV/s. The corrosion current density and the corrosion potential were determined from Tafel fitting. The experiments were carried out several times for standard statistical data processing. For data with good reproducibility, where data overlap occurs, we used 3 measurements, for as cast alloys Ti_94_Fe_1_Cu_1_Sn_4_ and Grade 1. In the case of Ti_94_Fe_1_Cu_1_Sn_4_ (SLM), for different layers of the surface of the working electrode, due to different phase deviations in them, as well as different relaxation of internal mechanical stresses, the polarization curve data were reproduced 5 times. For electrochemical tests, the typical curves were chosen.

## 3 Results and discussion

**Fig. 3:**
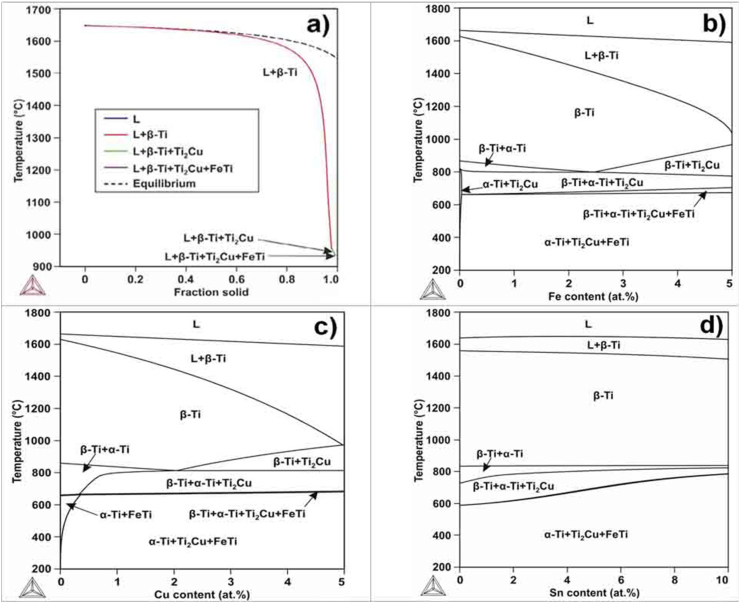
Equilibrium and non-equilibrium solidification pathway for the Ti_94_Fe_1_Cu_1_Sn_4_ alloy (a), the polythermal sections: Ti–4Sn–1Cu–Fe (b), Ti–4Sn–1Fe–Cu (c); Ti–1Cu–1Fe–Sn (d).

The equilibrium and non-equilibrium solidification of the Ti_94_Fe_1_Cu_1_Sn_4_ alloy was evaluated using the Thermo-Calc program. A graph showing the solidification pathway of the Ti_94_Fe_1_Cu_1_Sn_4_ alloy is presented in Fig. 3a. The dotted line (Fig. 3a) shows the solidification pathway at equilibrium solidification and in accordance with calculations, with the equilibrium solidification range of the alloy being 1649-1544 °C.

The obtained solidification range is short and differs slightly from the one obtained for titanium alloys Grade 1, Ti–6Al–4V [33,34] widely used for 3D printing. However, as shown by the solid lines in Fig. 3a, when non-equilibrium solidification proceeds, according to the Scheil-Gulliver [35] model, the solidus temperature of the alloy decreases to 950 °C and the formation of Ti_2_Cu and FeTi phases together with β-Ti occurs. In a real solidification process, the solidus is in a range of 1544–950 °C, and possibly closer to the lower value of the specified range due to the high cooling rate typical of SLM. This indicates that the Ti_94_Fe_1_Cu_1_Sn_4_ alloy has a long solidification range, which resulted in the technical complexity of obtaining high-quality casting or a molten track. To analyze the effect of the alloying elements on the solidification range, the polythermal sections of the Ti–Sn–Fe–Cu phase diagram were calculated, (Fig. 3b–d).

**Fig. 4:**
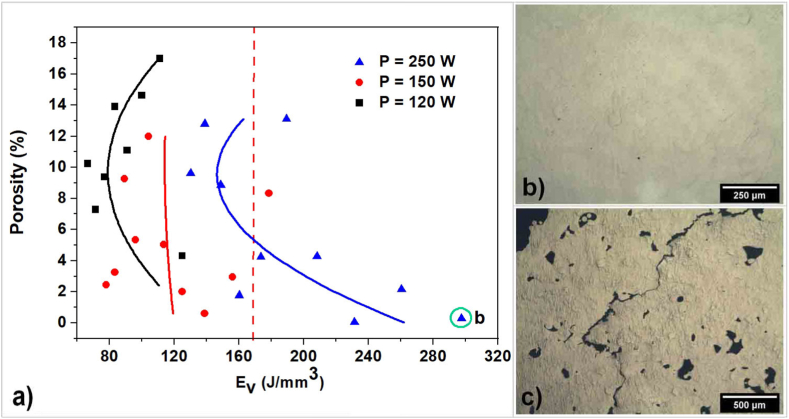
Effect of bulk energy density on porosity and hot tearing tendency (a), transverse structure morphology at E_v_ = 297.62 J/mm^3^ with porosity 0.4 % (b), transverse structure morphology at E_v_ = up to 156.25 J/mm^3^ with porosity up to 17.2 % and hot cracks (c).

In accordance with the polythermal section where the Sn content is varying, the solidus line is almost horizontal; and Sn cannot affect the solidification range of the alloy. However, Cu and Fe greatly increase the solidification range. Alloys with long solidification range are prone to the formation of hot cracks during solidification because such an alloy spends a longer time in the vulnerable state in which thin liquid films exist between the dendrites [36]. During SLM process that films can lead to the formation of defects, which were identified in the search for the optimal melting regime (Fig. 4b).

The power of laser, P (W), scanning speed, υ (mm/s), hatch distance, h (mm), and layer thickness, L_t_ (mm) were used to calculate the volumetric energy density E_v_ (J/mm^3^) [37,38], as a critical parameter used to determine optimal processing conditions:

(1) $$E_{v}=\frac{P}{\upsilon L_{t}h}\text{.}$$

From the dependences showing the influence of the volumetric energy density E_v_ on porosity, presented in Fig. 4a, we found that in the range E_v_ 62.5–156.25 J/mm^3^, hot cracks are formed (Fig. 4c), which increase defects when combined with pores, and at E_v_, more than 170 J/mm^3^, the absence of cracks is achieved for the regime at a laser power of 250 W with a decrease in porosity with an increase in E_v_.

It is known [39] that solidification hot tearing occurs in the late stages of solidification when the volume fraction of solid is above 85–95 %, and the solid phase is organized in a continuous network of grains. Resistance to hot tearing, as is known [39], is determined by three factors: brittleness temperature range, plasticity in this temperature range, and strain growth rate [39]. In this regard, the strain growth rate at a laser power of 250 W either contributes to sufficient relaxation of internal stresses or does not reach a value preceding cracking. In this case, with a decrease in the scanning speed and, accordingly, an increase in E_v_, the porosity decreases.

**Fig. 5:**
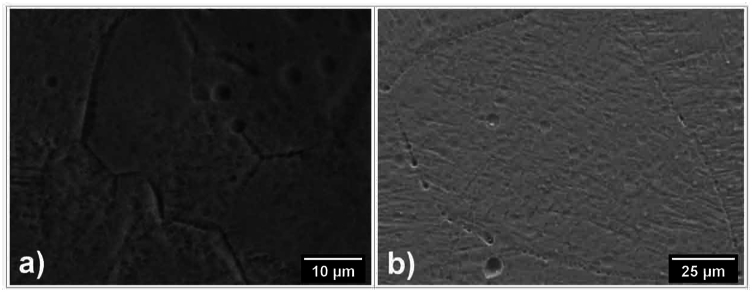
SEM images of the microstructure of the Ti_94_Fe_1_Cu_1_Sn_4_ alloy: after SLM (a); as-cast (b).

The grain size significantly decreases after SLM according to the optimal fusion mode (Ev = 297.62 J/mm^3^) and is about 20–30 μm (Fig. 5a), which coincides with the layer thickness in 3D printing. This is about 5 times less than for samples obtained by casting - the grain size is about 100 μm (Fig. 5b), which should potentially improve mechanical properties without subsequent heat treatment.

**Fig. 6:**
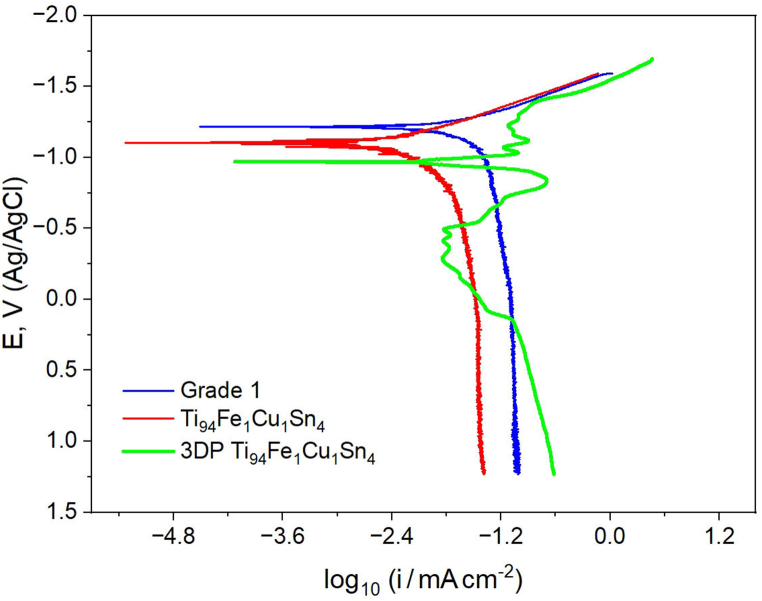
Electrochemical behavior of the Ti samples in HBSS at 37 °C. Potential - E, V (Ag/AgCl). Current density – log_10_(i/mA·cm^−2^).

To determine the electrochemical behavior of the Ti_94_Fe_1_Cu_1_Sn_4_ alloy after SLM, potentiodynamic curves were obtained. Fig. 6 shows the electrochemical behavior in HBSS at 37 °C of the Ti_94_Fe_1_Cu_1_Sn_4_ alloy after optimal SLM treatment, and as a comparison: Grade 1, Ti_94_Fe_1_Cu_1_Sn_4_ (as-cast). All samples are in the passive state and the value of the corrosion current density is low, as shown in Table 2. Ti Grade 1, the Ti_94_Fe_1_Cu_1_Sn_4_ (as-cast) samples have a more negative corrosion potential (Fig. 6, Table 2), whereas the anodic and cathodic curves of the Ti_94_Fe_1_Cu_1_Sn_4_ alloy sample after SLM have significant deviations from the cathodic to transpassive region. A possible reason for this behavior is the different microsegregation of the alloy components in as-cast and SLM conditions.

**Table 2:** Corrosion parameters of the titanium alloys: Grade 1 and Ti_94_Fe_1_Cu_1_Sn_4_ (in two states).

Alloy	Corrosion potential, V (Ag/AgCl)	Corrosion current density, μA/cm^2^	Corrosion current density in passive state, μA/cm^2^		
			0 mV	500 mV	1000 mV
As-cast Ti_94_Fe_1_Cu_1_Sn_4_	−1.107	4.58	31.85	36.24	37.61
Ti Grade 1	−1.209	9.91	80.04	84.94	87.56
Ti_94_Fe_1_Cu_1_Sn_4_ (SLM) E_v_ = 297.62 J/mm^3^	−0.97	12.97	36.83	128.12	204.52

As is known, titanium alloys are not subject to electrochemical corrosion in neutral media due to the formation of a protective passive film on the surface [[40], [41], [42], [43]]. As a result of our comparative analysis with similar curves for Grade 1, Ti_94_Fe_1_Cu_1_Sn_4_ alloys, it was found that due to the different protective ability of passive films on the studied samples, they have different inhibition of the anodic process.

As can be seen from Fig. 6 and Table 2, for Grade 1, as-cast Ti_94_Fe_1_Cu_1_Sn_4_, and the Ti_94_Fe_1_Cu_1_Sn_4_ alloy after SLM, different passive currents are observed. So, for example, at a potential of 0 mV, the currents in the passive state for Grade 1, as-cast Ti_94_Fe_1_Cu_1_Sn_4_ and the Ti_94_Fe_1_Cu_1_Sn_4_ alloy after SLM are: 80.04; 31.85; 36.83 μA/cm^2^, respectively. At this potential, the Ti_94_Fe_1_Cu_1_Sn_4_ alloy after SLM shows a similar behavior. However, at the potential of 500 mV, the currents in the passive state change significantly: as-cast Ti_94_Fe_1_Cu_1_Sn_4_ 36.24 μA/cm^2^; Ti_94_Fe_1_Cu_1_Sn_4_ after SLM to 128.12 μA/cm^2^ and increase for the Ti_94_Fe_1_Cu_1_Sn_4_ sample after SLM to 204.52 μA/cm^2^ at polarization up to 1000 mV.

Previously, the authors of [44] carried out electrochemical studies of the passive state of a Ti–5Cu binary alloy with different Ti_2_Cu phase sizes and Cu solid solution contents after heat treatments. As a result of their research, they found that with a decrease in the content of the Cu solid solution and an increase in the nano-sized Ti_2_Cu phase as a result of heat treatment (T6), the current in the passive state increases and fluctuates, which is consistent with the data we obtained for Ti_94_Fe_1_Cu_1_Sn_4_ (SLM) - a similar course of the curve. At the same time, in the work of the authors [44], it is also indicated that a further decrease in the Cu solid solution and an increase in the size of the Ti_2_Cu phase to a microsize leads to a decrease in the current and the absence of its fluctuations in the passive state. Probably, the deviation of currents in the passive state for the Ti_94_Fe_1_Cu_1_Sn_4_ alloy after SLM is also due to the heterogeneity of the chemical composition, size and distribution of the Ti_2_Cu phase, which further requires a search for heat treatment modes and a more in-depth study of the resulting passive films.

**Fig. 7:**
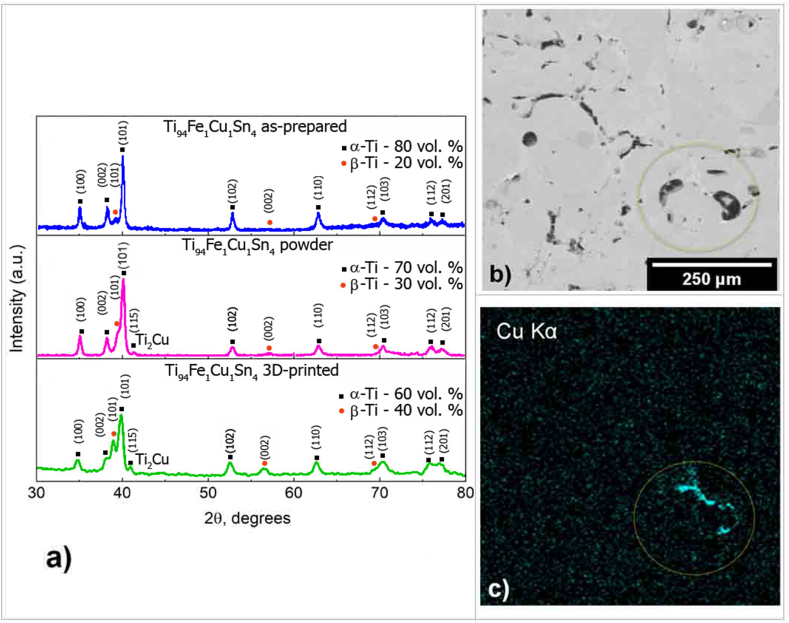
X-ray diffraction patterns of the Ti_94_Fe_1_Cu_1_Sn_4_ samples (a), obtained: via tilt-casting, sprayed to the powder, 3D printed. SEM images of an area of a sample with defects printed on a 3D printer (b) and mapping of the Cu element corresponding to this area (c).

As a result of the X-ray phase analysis, it was found that there are no significant differences between X-ray diffraction patterns of the as-cast samples (obtained via tilt-casting), the powder samples (obtained via sprayed), and 3D printed samples (printed from the sputtered powder), but the main proportion between α and β – Ti phases was changed (Fig. 7a). Also, in the element mapping picture of the samples obtained via sprayed and 3D printed, the segregation of the Cu element in some place of the interdendritic space is clearly seen (Fig. 7b,c), i.e., the samples have clearly detected microsegregation. As for the XRD analysis, it is not so clear, because there is only one weak peak, on the X-ray diffraction patterns of the sprayed and 3D printed samples, close to 42° 2θ, which probably corresponds to the Ti_2_Cu phase, but the amount of this intermetallic phase is not so significant (of about 1–2 vol %). In this case, the presence of copper segregations was established in result of the morphology analysis of the samples’ transverse structure at the periphery of the defects via scanning electron microscopy (Fig. 7c).

In accordance with the calculations of non-equilibrium solidification according to the Scheil-Gulliver model, the formation of Ti_2_Cu phase precipitates by eutectic transition in a small amount is possible (Fig. 3a). The probable presence of the Ti_2_Cu phase determines the unstable electrochemical behavior of the alloy after SLS in the Hanks solution (Fig. 6), as the Ti_2_Cu phase is an anodic structural component with respect to the base alloy [45]. Its presence leads to the cyclic disinhibition of the anode process with a release of Cu^2+^ ions. It was previously established that the corrosion rate strongly depends on the morphology of the Ti_2_Cu phase [46]. Based on our calculations (Fig. 3c), during homogenizing heat treatment at temperatures above 1000 °C, this phase should completely dissolve in the β-Ti solid solution. Also, during the subsequent search for the mode, the second stage of heat treatment is needed, it is desirable to achieve an optimal ratio of the α and β-Ti phases of 70:30 in terms of mechanical properties [22] without deteriorating the electrochemical behavior. At the same time, it is known that the presence of the Ti_2_Cu phase in binary titanium alloys of the Ti–Cu system has an excellent effect on antibacterial properties [46,47]. Furthermore, with the acicular Ti_2_Cu phase, the antibacterial effect is two times higher than with the presence of the finely dispersed Ti_2_Cu phase. The acicular precipitate has a higher Cu:Ti ratio, resulting in an increased release of Cu^2+^ ions. In this regard, it is advisable in further studies to also determine the dependence of the size factors of the distribution of the Ti_2_Cu phase in the volume of the Ti_94_Fe_1_Cu_1_Sn_4_ alloy on the corrosion-mechanical properties.

## 4 Conclusion

This paper studied the potential possibility of selective laser melting of a new alpha-beta-titanium alloy Ti_94_Fe_1_Cu_1_Sn_4_, which has high mechanical characteristics and does not contain components that cause allergic reactions [23], with the aim of further research to manufacture personalized implants of a new generation. The spherical powders of the Ti_94_Fe_1_Cu_1_Sn_4_ alloy were obtained by plasma atomization, from which the optimal regime for obtaining samples was determined by the SLM method. It was found that when the volume energy density E_v_ is less than 173.61 J/mm^3^, hot cracks are formed, and an increase in E_v_ to 297.62 J/mm^3^ reduces the porosity of the samples to 0.4 %. Based on the results of the calculation of non-equilibrium solidification, as well as the study of morphology and X-ray phase analysis, it was established that the non-equilibrium Ti_2_Cu phase may precipitate out, which, being an anodic structural component, changes the electrochemical behavior of the Ti_94_Fe_1_Cu_1_Sn_4_ alloy. Taking into account the significant influence of the size factors of the distribution of the Ti_2_Cu phase on the corrosion process and its favorable effect on antibacterial properties, recommendations were made to continue research on the effect of heat treatment modes on the mechanical and corrosion properties of samples after SLM. It is advisable, in addition to classical heat treatment, to conduct a study on the effect of hot isostatic pressing (HIP). From the point of view of subsequent deformation processing, the TiFeCuSn alloy has good prospects because it is known [48] that the increased the solidification range that promotes grain refinement due to increasing of segregating power [49], the finer the initial cast grain will be, which is useful for achieving superplasticity [50].

## Data availability statement

Data will be made available on request.

## Additional information

No additional information is available for this paper.

## CRediT authorship contribution statement

**V.A. Bautin:** Writing – original draft, Resources, Project administration, Investigation. **V. Yu Zadorozhnyy:** Writing – review & editing, Funding acquisition, Conceptualization. **A.A. Korol:** Methodology, Investigation, Formal analysis. **V.E. Bazhenov:** Investigation, Formal analysis, Data curation. **A.S. Shinkarev:** Validation, Methodology, Investigation, Data curation. **S.V. Chernyshikhin:** Visualization, Validation, Methodology, Investigation, Data curation. **D.O. Moskovskikh:** Resources, Formal analysis, Data curation. **M.E. Samoshina:** Writing – review & editing, Data curation. **A.A. Khort:** Writing – review & editing, Investigation, Funding acquisition.

## Declaration of competing interest

The authors declare that they have no known competing financial interests or personal relationships that could have appeared to influence the work reported in this paper.
